# Imaging the Centromedian Thalamic Nucleus Using Quantitative Susceptibility Mapping

**DOI:** 10.3389/fnhum.2019.00447

**Published:** 2020-01-09

**Authors:** Jun Li, Yufei Li, Lorenzo Gutierrez, Wenying Xu, Yiwen Wu, Chunlei Liu, Dianyou Li, Bomin Sun, Chencheng Zhang, Hongjiang Wei

**Affiliations:** ^1^Department of Functional Neurosurgery, Ruijin Hospital, Shanghai Jiao Tong University School of Medicine, Shanghai, China; ^2^Institute for Medical Imaging Technology, School of Biomedical Engineering, Shanghai Jiao Tong University, Shanghai, China; ^3^Department of Neurology, Ruijin Hospital, Shanghai Jiao Tong University School of Medicine, Shanghai, China; ^4^Department of Electrical Engineering and Computer Sciences, University of California, Berkeley, Berkeley, CA, United States; ^5^Helen Wills Neuroscience Institute, University of California, Berkeley, Berkeley, CA, United States

**Keywords:** deep brain stimulation, direct targeting, gradient recalled echo, quantitative susceptibility mapping, centromedian nucleus

## Abstract

The centromedian (CM) nucleus is an intralaminar thalamic nucleus that is considered as a potentially effective target of deep brain stimulation (DBS) and ablative surgeries for the treatment of multiple neurological and psychiatric disorders. However, the structure of CM is invisible on the standard T1- and T2-weighted (T1w and T2w) magnetic resonance images, which hamper it as a direct DBS target for clinical applications. The purpose of the current study is to demonstrate the use of quantitative susceptibility mapping (QSM) technique to image the CM within the thalamic region. Twelve patients with Parkinson’s disease, dystonia, or schizophrenia were included in this study. A 3D multi-echo gradient recalled echo (GRE) sequence was acquired together with T1w and T2w images on a 3-T MR scanner. The QSM image was reconstructed from the GRE phase data. Direct visual inspection of the CM was made on T1w, T2w, and QSM images. Furthermore, the contrast-to-noise ratios (CNRs) of the CM to the adjacent posterior part of thalamus on T1w, T2w, and QSM images were compared using the one-way analysis of variance (ANOVA) test. QSM dramatically improved the visualization of the CM nucleus. Clear delineation of CM compared to the surroundings was observed on QSM but not on T1w and T2w images. Statistical analysis showed that the CNR on QSM was significantly higher than those on T1w and T2w images. Taken together, our results indicate that QSM is a promising technique for improving the visualization of CM as a direct targeting for DBS surgery.

## Introduction

The centromedian nucleus (CM) or centromedian–parafasicular nucleus complex, located in the caudal intralaminar thalamic nuclei, has been reported to be a potentially effective target for deep brain stimulation (DBS) or ablative surgeries for the treatment of various neurological and psychiatric diseases, e.g., Parkinson’s disease, Tourette syndrome, generalized epilepsy, and intractable neuropathic pain (Ilyas et al., 2019). However, the surgeries targeting CM still relied on the indirect targeting method by registering a normalized atlas to the patient’s magnetic resonance imaging (MRI) data and then the CM coordinates are used for target localization (Krauss et al., 2002; Kim et al., 2017; Sharma et al., 2017). This indirect targeting method may lead to suboptimal targeting since significant variations exist in brain structures between patients, and this variation causes unpredictable registration errors (Kennedy et al., 1998) and may sub-optimize treatment effect and increase the rate of surgical complications and adverse side effects (Chan et al., 2009).

Direct targeting can improve the targeting accuracy in certain aspects as revealed by some studies (Tonge et al., 2016; Fenoy and Schiess, 2018). Direct targeting requires that the anatomical locations can be visible on certain image contrast. However, direct visualization of the CM nucleus using the standard T1w and T2w MRI sequences is challenging. On one hand, the volume of the CM is small (smaller than 10 mm in most dimensions; Ilyas et al., 2019). On the other hand, the contrast between the CM nucleus and its surrounding thalamic structures is pretty low. The absence of an imaging technique for direct visualization of CM hampers the targeting accuracy of CM for DBS surgery.

Some researchers have made considerable efforts to improve the individualized depiction of thalamic substructures. Lemaire et al. (2010) reported that high-resolution T1w images could be used to image the substructures of the thalamus, which were very comparable to myelin-stained histologic sections. However, the scan time for the protocol was approximately 14 h, which is not suitable for routine clinical scans. Kanowski et al. (2010) showed that the CM is identifiable in a reasonable measurement time of 13–26 min with two-dimensional high-resolution proton-attenuation-weighted images at 3 T. However, only a few slices in axial plane covering the localized areas were acquired, which still challenges targeting localization when using the surgical planning software involving the 3D image registration procedure. Bender et al. (2011) demonstrated that the CM could be roughly identified by optimized 3D MPRAGE protocol, which would take about 20 min to be acquired; however, clear discrimination of all thalamic substructures were not achievable. If anatomic imaging-based targeting methods can be further improved, the accuracy and efficiency of target selection for DBS or ablative surgeries may further increase.

Quantitative susceptibility mapping (QSM) reconstructed from the MRI phase images of the 3D gradient recalled echo (GRE) sequences could improve tissue contrast compared to T2w images. QSM employed deconvolution of GRE phase images and removed the non-local susceptibility effects, depicting more accurate structural delineation (Liu et al., 2015). QSM has been clinically used to assess important tissue functions and disease (Wang et al., 2017), and recently it has been demonstrated for improving the depiction of DBS target structures with iron-rich nucleus (paramagnetic), e.g., the subthalamic nucleus (Liu et al., 2013; Alkemade et al., 2017) and the globus pallidus internus (Wei et al., 2019), with the surrounding white matters (diamagnetic). The thalamus contains different subregions that are known to have various iron deposits and different degrees of myelinated white matters (Morris et al., 1992; Zhang et al., 2018), which indicates that QSM, by using the susceptibility differences existing between substructures, may be a proper imaging technique to identify CM.

The aim of this study is to examine whether QSM could delineate the CM nucleus from its adjacent thalamic structures and thus generate a direct visualization of the CM.

## Materials and Methods

### Human Subjects

Twelve patients (six males and six females, mean age 41.8 ± 21.2 years old) with Parkinson’s disease (*n* = 5, mean age 61.0 ± 16.6), dystonia (*n* = 4, mean age 32.8 ± 8.6), or schizophrenia (*n* = 3, mean age 21.7 ± 10.3) were included as convenient samples in this study. Demographic information collection and neuroradiological investigation were performed by specialized movement disorder neurologists or psychiatrists. The study was approved by the ethics committee of Ruijin Hospital, School of Medicine, Shanghai Jiao Tong University. All subjects provided written consent in accordance with the Declaration of Helsinki.

### Data Acquisition

Imaging was performed on a 3.0-T MR scanner equipped with a 24-channel head coil. Each subject lay supine with their head snugly fixed with foam pads. The subject was asked to keep still as long as possible. 3D T1w and axial T2w images were acquired. A multi-echo GRE sequence was also performed. Detailed imaging parameters, including the time of repetition, time of echo, field of view, voxel size, and total duration of scanning for the three imaging modalities, are summarized in Table 1.

**Table 1:** Imaging parameters.

Parameter	3D T1w	2D T2w	3D GRE
Imaging plane	Axial	Axial	Axial
Field of vision (mm)	240 × 240	240 × 240	240 × 240
Matrix	320 × 320	320 × 320	320 × 320
Resolution (mm)	0.75 × 0.75 × 1.5	0.75 × 0.75 × 1.5	0.75 × 0.75 × 1.5
Time of repetition (ms)	7.04	3,000/4,000	32.80
Time of echo (ms)	3.47	128.60/106.03	11.00
Scan time (s)	172	346	528

### Image Processing

QSM images were reconstructed from GRE phase data. The details of QSM processing has been documented in the previous articles (Wei et al., 2015, 2017). In brief, three major steps were taken for the reconstruction of the QSM image. First, the phase images of GRE were unwrapped using a Laplacian-based phase unwrapping. Afterward, the magnitude images were used to extract the brain tissue using the FMRIB Software Library Brain Extraction Tool^1^. Then, the background phases were removed using the V_SHARP method to obtain the local tissue phase images (Li et al., 2015). Finally, susceptibility maps were generated after dipole inversion using streaking artifact reduction for QSM method (STAR-QSM; Wei et al., 2015).

### Image Inspection and Data Analysis

Firstly, we compared the QSM images to a schematic drawing referenced from the overlay of Schaltenbrand and Wahren histologic atlas (Schaltenbrand et al., 1977) to confirm whether the CM can be visible on the QSM image. To calculate the contrast-to-noise ratios (CNRs), QSM and T2w images were firstly registered to the T1w image. Then, the regions of CM and the adjacent posterior thalamic tissues were manually defined as masks on the QSM image (Supplementary Figure S1). Afterward, the masks of CM and posterior thalamus were applied to the T1w and T2w images. The CNRs of the CM nucleus referenced to the posterior thalamus were measured: CNR = |S_CM_−S_pTH_|/σ, where S_CM_ and S_pTH_, respectively, represent the mean signal intensities of the CM nucleus and posterior part of thalamus. σ represents noise measurement calculated as the standard deviation of the signal intensities in the posterior part of thalamus. The volumes of the CM nucleus were also calculated on QSM images, by multiplying the number of CM voxels and the voxel size.

### Statistical Analysis

A one-way analysis of variance (ANOVA) was used to compare the difference in CNRs among the three MR image modalities (IBM SPSS Statistics, version 22). If the one-way ANOVA gave a significant result, independent two-sample *t*-tests were further used as the *post hoc* tests to reveal the CNR differences between each two modalities (T1w vs. T2w, T1w vs. QSM, and T2w vs. QSM). Two-way repeated-measure ANOVAs were also performed to examine the significance of interaction between image modality (T1w, T2w, and QSM) and patient type (Parkinson’s disease, dystonia, and schizophrenia), and the significance of interaction between CM volume (left CM and right CM volumes) and patient type. The threshold of significance was set at *p* < 0.05.

## Results

**Figure 1:**
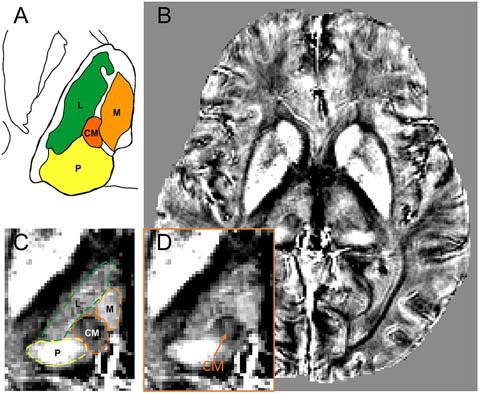
The visualization of CM within the thalamus on quantitative susceptibility mapping (QSM) image. **(A)** A schematic drawing of the CM and its surrounding thalamic structures, referenced to the overlay of the Schaltenbrand and Wahren atlas (Schaltenbrand et al., 1977). **(B)** An axial view of a slice of QSM image with thalamic substructures on a representative patient. **(C)** Enlarged view of thalamic substructures with the anatomical boundaries of CM and its surrounding thalamic parts (medial, lateral, and posterior) delineated. **(D)** Enlarged view of thalamic substructures. The anatomical location of CM nucleus is pointed by an orange arrow. Abbreviations: CM, centromedian nucleus. L, lateral part of thalamus; M, medial part of thalamus; P, posterior part of thalamus.

Figure 1A shows a schematic drawing of thalamus that is referenced to the Schaltenbrand and Wahren atlas (Schaltenbrand et al., 1977). The diamagnetic CM is surrounded by the relatively paramagnetic medial, lateral, and posterior parts of the thalamus (Figure 1A). Figures 1B,D show the QSM image of one representative patient. As shown, the QSM image provides a clear visualization on the anatomical structure of the CM (as indicated by an orange arrow) in a patient with Parkinson’s disease. The anatomical boundaries of the medial, lateral, and posterior parts of the thalamus are also visible owing to different magnetic susceptibility values, as delineated in Figure 1C.

**Figure 2:**
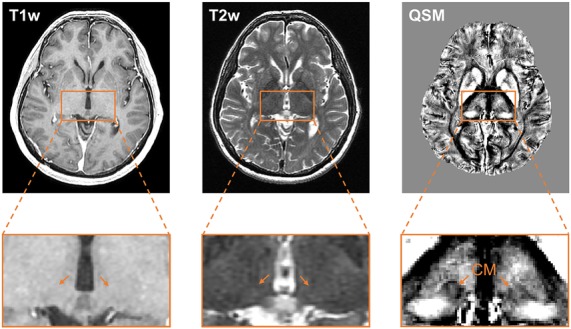
Comparison of the visualization of the CM nucleus on T1w, T2w, and QSM images. Axial slice views (upper row) and enlarged views of the thalamus (lower row) on T1w, T2w, and QSM images at one representative section on a representative patient. Abbreviations: CM, centromedian nucleus; QSM, quantitative susceptibility mapping.

Figure 2 compares the contrast of CM on T1w, T2w, and QSM images at one representative section of a representative patient. The location of CM nucleus is difficult to be identified on the T1w or T2w images. However, QSM image clearly shows the substructures of the thalamus, for example, medial, lateral, and posterior parts of the thalamus. The CM nucleus is delineable from its surroundings on the QSM image. Clear delineation of CM and the surrounding tissues is attributed to the susceptibility difference existed between iron-rich nucleus and the adjacent myelinated white fiber axons. The QSM image exhibits a diamagnetic susceptibility within the CM and a relatively paramagnetic susceptibility of its surrounding thalamic tissues. The T1w, T2w, and QSM images at one representative section containing CM nucleus on each patient are presented in the Supplementary Figure S2.

**Figure 3:**
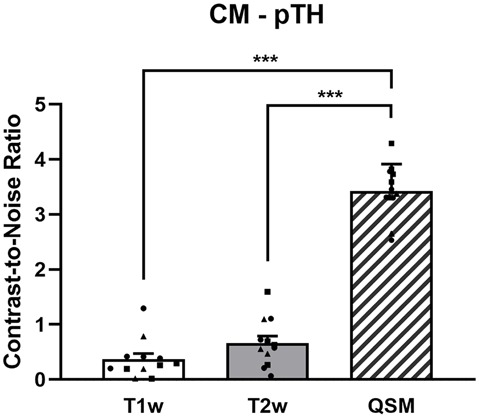
The CNRs of the CM to the posterior part of thalamus on the T1w, T2w, and QSM images. The dots represent the individual values of the Parkinson’s disease patients (square dots), the dystonia patients (circular dots), and the schizophrenia patients (triangle dots). ***Indicates *p* < 0.001. Abbreviations: CM, centromedian nucleus; pTH, posterior thalamus; QSM, quantitative susceptibility mapping.

The CNRs of the CM nucleus to the posterior part of thalamus are 0.37 ± 0.35, 0.67 ± 0.43, and 3.43 ± 0.49, respectively, on T1w, T2w, and QSM images (Figure 3). The ANOVA reveals significant differences among T1w, T2w, and QSM images in terms of the CNR, *F*_(2)_ = 177.14, *p* < 0.001 (Figure 3). *Post hoc* tests (independent two-sample *t*-tests) indicate significant different CNRs between QSM and T1w (*t*_(11)_ = 16.66, *p* < 0.001), and between QSM and T2w (*t*_(11)_ = 17.44, *p* < 0.001). The mean CNRs for each type of patients are illustrated in the Supplementary Table S1, in which increased CNRs on QSM images are indicated in each of the three patient types. The mean volumes of the left and right CM nuclei are 160.95 ± 29.98 mm^3^ and 169.73 ± 50.34 mm^3^, respectively, as detected on the QSM images. No significant main effects of patient type on CNR value or CM volume, or interactions between patient type and CNR value, or between patient type and CM volume were found in our sample (*p*s > 0.142, Supplementary Tables S1, S2).

## Discussion

The results demonstrate that with the QSM technique, the CM can be clearly delineated from the surrounding subthalamic nuclei. Compared with commonly used T1w and T2w images for DBS planning, QSM significantly improved the CNR of CM nucleus compared to its surrounding thalamic structures, suggesting that a QSM-based image is more suitable to target the patient-specific CM in DBS surgery directly.

Aside from the surgical targets routinely used in clinical treatment (e.g., subthalamic nucleus, nucleus accumbens), there are some other targets with potential effectiveness in treating neurological and psychiatric diseases. The CM nucleus or centromedian–parafasicular nucleus complex, situated within the intralaminar nuclei of the thalamus, has abundant fiber connections with other thalamic nuclei, basal ganglia, and cerebral cortex (Ilyas et al., 2019). In several studies, the CM nucleus has been suggested as a potentially effective DBS target for the treatment of Parkinson’s disease (Caparros-Lefebvre et al., 1999; Mazzone et al., 2006; Peppe et al., 2008; Stefani et al., 2009) and Tourette syndrome (Houeto et al., 2005; Savica et al., 2012; Testini et al., 2016; Marano et al., 2019). The clinical surgeries targeting at CM also show treatment effect for the generalized epilepsy (Fisher et al., 1992; Velasco et al., 2007; Valentín et al., 2013; Li and Cook, 2018) and intractable neuropathic pain (Young et al., 1995; Hollingworth et al., 2017) by means of DBS or thalamotomy. The DBS surgery targeting the CM nucleus currently uses indirect ways in which a two-dimensional stereotactic atlas of the thalamus is superimposed on a CT or MRI scan relative to coarse anatomical landmarks including anterior and posterior commissures (Stefani et al., 2009; Son et al., 2016; Testini et al., 2016). The indirect method of targeting the CM nucleus is due to the small volume of the CM, measuring smaller than 10 mm in most dimensions (Ilyas et al., 2019), and low image contrast between the CM nucleus and its surrounding thalamic structures on conventional MRI images. The challenge of precisely locating the nucleus would limit the clinical application and the efficacy of CM-DBS. Inter-patient variability may affect the accuracy of the placement DBS electrodes, and may sub-optimize treatment effect and increase the rate of surgical complications and adverse side effects (Chan et al., 2009). Direct imaging CM can be of great help for direct targeting of this intralaminar thalamic nucleus.

Recently developed QSM image reconstructed from the GRE-sequence image is an effective technique that takes advantage of differentiated iron concentration in different subcortical microstructures to identify their locations (Deistung et al., 2017). Thalamic nuclei have sufficient iron concentration and different nuclei are with different levels of iron deposits (Drayer et al., 1986; Morris et al., 1992). Thus, QSM can delineate one nucleus from its adjacent myelinated white matter axons, such as for imaging the CM in this study. The delineation of CM is attributed to the susceptibility difference existed in iron concentration compared to the adjacent myelin sheath fibers. Although CM nucleus is also visible on high-resolution T1w images, 2-D proton-attenuation-weighted images, or images acquired by optimized 3D MPRAGE protocol (Kanowski et al., 2010; Lemaire et al., 2010; Bender et al., 2011), those images usually would take at least 20 min (or even hours) to be acquired. GRE image of the whole brain can be acquired within less than 10 min, which is more realistic for routine clinical scans for DBS planning.

Based on our finding that QSM could provide direct visualization on CM nucleus, together with the recent findings that QSM could also provide superior anatomical delineation in subthalamic nucleus (Liu et al., 2013; Alkemade et al., 2017) and globus pallidus internus (Wei et al., 2019), the implementation of QSM imaging in clinical settings for relevant diseases should be given consideration by radiologists, neurosurgeons, MR manufacturers, and engineers. On the other hand, the QSM technique has plenty of room to improve on for clinical applications, including shortening acquisition time and reducing streaking artifacts to further improve the image quality (Wang et al., 2017).

The signal intensity on a QSM image depends on the tissue magnetic susceptibility (Wang and Liu, 2015). Due to the rich abundancy of iron in the blood, the blood vessel on a QSM image has a much higher intensity than gray matter, white matter, or cerebrospinal fluid (Haacke et al., 2015). The visual identification of the CM nucleus in the present sample is unaffected by the blood vessels nearby. Furthermore, strong QSM signal can be observed in the structures with bleeding or vascular dysmorphia (Liu et al., 2012, 2015; Chen et al., 2014). Although not being observed in the individuals of our sample, the delineation of the thalamic structures, including the CM nucleus, could be blurred in individuals with micro-bleeding or vascular dysmorphia at or around the regions of interest.

There are some limitations in the current study. The 3D GRE sequences is quite sensitive to patients’ motion during the scan, and thus the application in patients with obvious tremor might be limited. The next limitation is that the scanning process for whole-brain QSM takes nearly 5–10 min. Although it is faster than the other methods that can also demonstrate the CM nucleus (Kanowski et al., 2010; Lemaire et al., 2010; Bender et al., 2011), more rapid QSM techniques are yet to be invented for DBS targeting in clinical application (Wei et al., 2019). Another limitation is that the segmentations were done manually in this study. In future studies, the QSM images could be normalized to MNI space and segmented based on available subcortical 3D atlases, e.g., using Lead-DBS toolbox^2^. Finally, the sample size of the present study is relatively small. However, even with small sample size, the superiority of QSM for depicting CM nucleus can still be observed. The negative results of CNR values and CM volumes between different types of patients may be attributed to the limited sample size. Future studies with large sample sizes are needed to reveal the profiles of CNR values and CM volumes in different types of patients, particularly in the patients where DBS has shown potential effectiveness (e.g., Parkinson’s disease, Tourette syndrome, generalized epilepsy, and intractable neuropathic pain).

## Conclusion

In summary, we have demonstrated that the QSM images provide a significantly clearer visualization of the CM nucleus than T1w and T2w images, suggesting that a QSM image is likely more suitable to aid directly determining patient-specific CM coordinates in the DBS and ablative surgeries. Future studies are highly needed to evaluate the QSM imaging CM nucleus on a large sample size, particularly in the types of patients who might potentially benefit from DBS treatment, and confirm whether QSM technique can improve DBS targeting accuracy or effectiveness compared with indirect targeting methods.

## Data Availability Statement

The datasets generated for this study are available on request to the corresponding author.

## Ethics Statement

The studies involving human participants were reviewed and approved by the ethics committee of Ruijin Hospital, School of Medicine, Shanghai Jiao Tong University. The patients/participants provided their written informed consent to participate in this study.

## Author Contributions

YW, DL, BS, CZ, and HW conceived and designed the study. WX and HW collected the data. JL, YL, LG, WX, and CL analyzed the data. JL, YL, LG, YW, CL, DL, BS, CZ, and HW interpreted the data and wrote the article.

## Conflict of Interest

The authors declare that the research was conducted in the absence of any commercial or financial relationships that could be construed as a potential conflict of interest.

## Footnotes

^1^https://fsl.fmrib.ox.ac.uk/fsl/fslwiki/BET

^2^www.lead-dbs.org

## Abbreviations

CM: centromedian nucleus
CNR: contrast-to-noise ratio
DBS: deep brain stimulation
GRE: gradient recalled echo
MRI: magnetic resonance imaging
QSM: quantitative susceptibility mapping
T1w: T1-weighted
T2w: T2-weighted.
