# Supplementary material for: Imaging the Centromedian Thalamic Nucleus Using Quantitative Susceptibility Mapping
Source: Front Hum Neurosci. 2020 Jan 9;13:447. doi: 10.3389/fnhum.2019.00447 (PMC6962173; doi:10.3389/fnhum.2019.00447)
Supplement: Supplementary file 1 [file Data_Sheet_1.PDF]

Table S1. Mean CNR values on T1w, T2w and QSM images in each type of patients.

CNR values (mean±SD)	T1w	T2w	QSM	Statistics (repeated-measure ANOVA)		
				Patient Type	Image Modality	Interaction
**PD (n=5)**	0.54±0.43	0.54±0.42	3.28±0.46	$F(2,18)=0.959$ $p=0.419$	$F(2,18)=198.21$ $p<0.001$	$F(4,18)=1.972$ $p=0.142$
**Dys (n=4)**	0.19±0.12	0.80±0.57	3.86±0.30			
**Sch (n=3)**	0.33±0.40	0.71±0.34	3.11±0.40			

ANOVA, analysis of variance; Dys, dystonia; PD, Parkinson's disease; Sch, schizophrenia.

Table S2. Mean volumes of CM detected from QSM images in each type of patients.

CM volumes (mean±SD)	Left CM (mm^3)	Right CM (mm^3)	Statistics (repeated-measure ANOVA)		
			**Patient Type**	**Laterality (left vs. right)**	**Interaction**
**PD (n=5)**	166.1±27.4	188.0±39.3	F(2,9)=0.335 p=0.724	F(1,9)=0.848 p=0.381	F(2,9)=1.104 p=0.372
**Dys (n=4)**	157.4±38.2	155.0±68.8			
**Sch (n=3)**	157.2±33.8	158.9±48.0			

ANOVA, analysis of variance; Dys, dystonia; PD, Parkinson’s disease; Sch, schizophrenia.

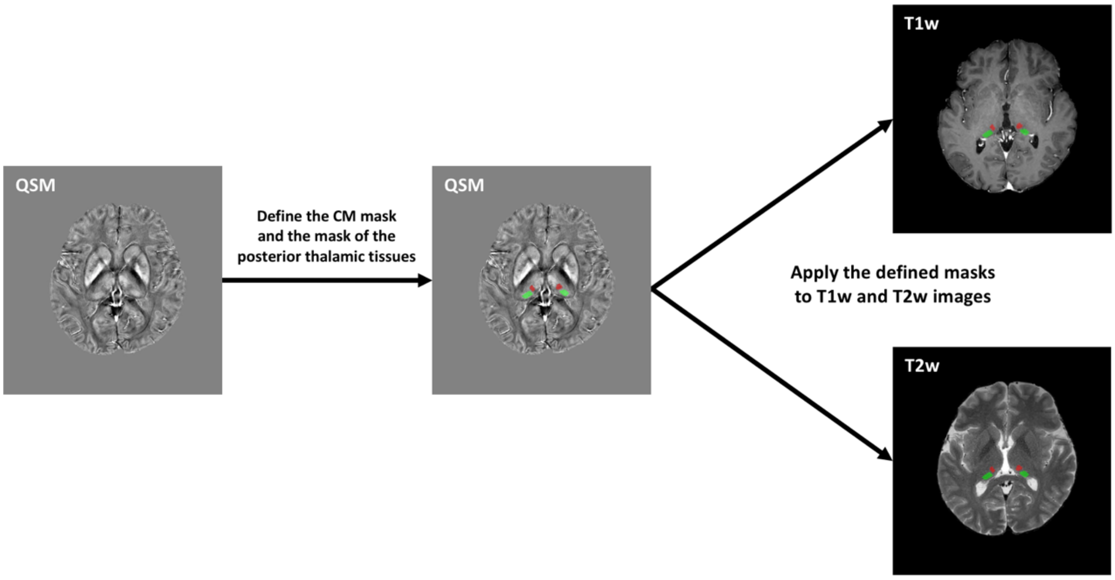

Figure S1. Masks of the CM and the posterior part of thalamus. The CM and the posterior part of thalamus were manually defined on a representative section of the QSM image as the masks for CNR calculation. The masks were then applied to the T1w and T2w images for the calculation of CNR. The red clusters indicate the masks of bilateral CM nuclei, and the green clusters represent the masks of bilateral posterior thalamic tissues.

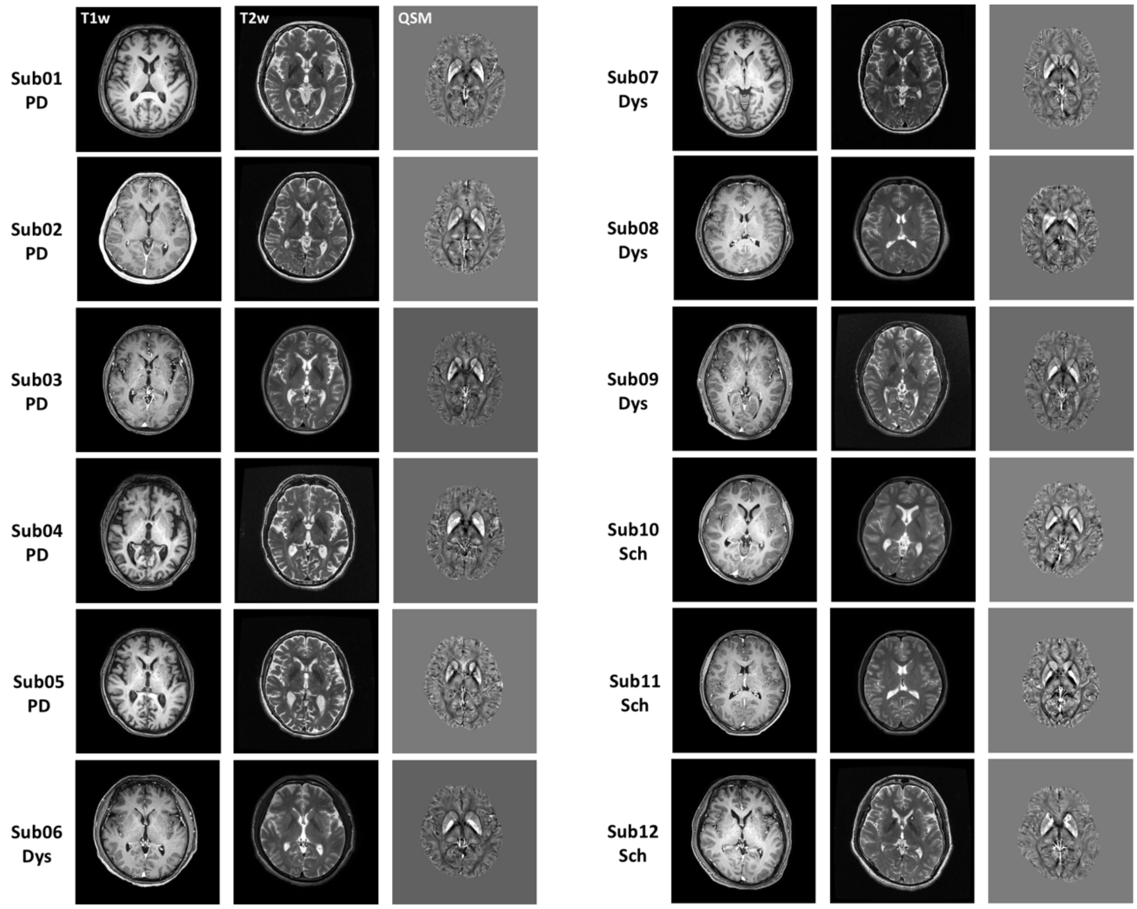

Figure S2. T1w, T2w and QSM images at one representative section containing CM nucleus on each patient. Dys, dystonia; PD, Parkinson's disease; Sch, schizophrenia; Sub, subject.
